# On Designing Thermal-Aware Localized QoS Routing Protocol for *in-vivo* Sensor Nodes in Wireless Body Area Networks

**DOI:** 10.3390/s150614016

**Published:** 2015-06-15

**Authors:** Muhammad Mostafa Monowar, Fuad Bajaber

**Affiliations:** Department of Information Technology, Faculty of Computing and Information Technology, King AbdulAziz University, Jeddah-21589, Saudi Arabia; E-Mail: fbajaber@kau.edu.sa

**Keywords:** localized routing, wireless body area networks, thermal-aware, in-vivo sensors

## Abstract

In this paper, we address the thermal rise and Quality-of-Service (QoS) provisioning issue for an intra-body Wireless Body Area Network (WBAN) having *in-vivo* sensor nodes. We propose a thermal-aware QoS routing protocol, called TLQoS, that facilitates the system in achieving desired QoS in terms of delay and reliability for diverse traffic types, as well as avoids the formation of highly heated nodes known as hotspot(s), and keeps the temperature rise along the network to an acceptable level. TLQoS exploits modular architecture wherein different modules perform integrated operations in providing multiple QoS service with lower temperature rise. To address the challenges of highly dynamic wireless environment inside the human body. TLQoS implements potential-based localized routing that requires only local neighborhood information. TLQoS avoids routing loop formation as well as reduces the number of hop traversal exploiting hybrid potential, and tuning a configurable parameter. We perform extensive simulations of TLQoS, and the results show that TLQoS has significant performance improvements over state-of-the-art approaches.

## 1. Introduction

Thanks to Moore's law, the continuous decrease in size while growing capacity increase of electronic devices has made it inevitable for the development of tiny and portable devices that can communicate with each other around the human body. This substantial development creates growing interest amongst researchers, system designers and application developers on a new type of network architecture generally known as a Wireless Body Area Network (WBAN). A WBAN is thus a special type of network that integrates miniaturized, intelligent, low-power sensor nodes in, on or around a human body that continuously monitor the body functions and surrounding environment [1].

WBAN is equipped with nodes that are wearable and/or implanted, and capable of wireless communication to transmit data to a nearby coordinator known as the Body Coordinator (BC). An implanted sensor node, also known as an *in-vivo* node is a special type of sensor which detects and collects the desired biometric data of a certain physiological change inside the body, and transmits the data to the BC. One of the major problems caused by continuous sensing of *in-vivo* sensor node is the heat produced due to wireless communication and the power dissipation by the sensor circuitry [2,3]. The increased heat causes thermal damage to the human tissue inside the body if the communication is prolonged for a long time which might be a threat to the human life [4]. Hence, temperature rise in one the significant issues to be considered in designing communication protocols for WBAN having *in-vivo* nodes.

Because of the high potential in e-healthcare, WBAN has been facilitated nowadays with wider range of applications [5] having distinct Quality of Service (QoS) demands in terms of delay, reliability, throughput *etc.* Moreover, a patient can be equipped with diverse *in-vivo* sensors with various QoS requirements. For instance, electroencephalogram (EEG), electrocardiogram (ECG) and Electromyography (EMG) *etc.* require higher reliability with real time delivery. However, the traffic from respiration monitoring, pH-level monitoring sensors demand higher reliability but can tolerate delay to some extent. Thus, addressing traffic heterogeneity satisfying respective QoS requirements is another considerable challenging issue for the design of WBAN communication protocols.

Although a number of implementations generally use a single-hop communication architecture for a WBAN to connect all sensors to a central sink node, recent researchers [6–10] pointed out that the multihop communication paradigm is more energy-efficient and even necessary when applied inside the human body with innate severe propagation loss. Considering the temperature rise as a striking metric, a number of studies presented thermal-aware communication protocols exploiting the multi-hop communication paradigm for WBAN having *in-vivo* nodes. These protocols mainly aim to minimize the temperature rise in route selection procedure and ignore the QoS issues. There are also few researches found in the literature considering QoS provisioning in WBAN but these studies neglect the thermal effect [11–13].

Considering the thermal-awareness as well as QoS provisioning with the support of traffic heterogeneity of implanted WBAN, recently TMQoS [14] has been proposed. TMQoS employs a cross-layer proactive routing framework that maintains an ongoing routing table containing end-to-end routes for all traffic types. Thus, this protocol requires not only local knowledge, but also the knowledge of end-to-end network information for the route selection towards the *BC*. Although TMQoS selects the best end-to-end route satisfying certain QoS parameters, but it has some drawbacks in the context of WBAN. First, this overhead to too high to scale the network to a good number of nodes. Secondly, the wireless environment inside the human body is highly dynamic due to its variability in path loss [15]. The situation becomes more significant in the presence of implant sensors with different QoS demands and data rates. This highly dynamic nature prolongs the network convergence time that could cause stale network information to the source nodes while selecting the route. Besides these drawbacks, TMQoS only considers the available shortest path routes for traffic dissemination. However, the shortest-path might not always be the best end-to-end path considering the desired QoS metrics and temperature rise.

In this paper, we present TLQoS, a Thermal-aware Localized QoS routing protocol for *in-vivo* sensor nodes in WBAN, which exploits a localized approach in route selection with the aim of satisfying the required QoS demands of diverse *in-vivo* nodes as well as preventing the formation of highly heated nodes, known as hotspot(s) along the network. TLQoS employs potential-based greedy routing approach that reflects its localized behavior requiring only local neighborhood information. TLQoS defines a number of routing potentials based on the QoS metrics and node temperature. To avoid the formation of routing loops which might be common in typical greedy approaches, and to route the packet towards the sink reducing unnecessary hop traversal, hybrid potentials are introduced, and a routing loop avoidance mechanism is presented in TLQoS. TLQoS exploits a completely modular approach to address the traffic according to their respective QoS demands. TLQoS effectively measures the parameters for route selection exploiting a cross-layer framework. Furthermore, the performance of TLQoS is evaluated compared to the state-of-the-art protocols using simulations.

The rest of the paper is organized as follows: Section 2 summarizes the related works. Section 3 presents the system model and states the preliminaries behind the protocol. Section 4 describes the proposed protocol. Section 5 demonstrates the protocol performance using simulation. Finally, Section 6 presents the concluding remarks.

## 2. Related Works

In recent times, considering thermal-effect on human body as one of the striking criteria, a series of communication protocols have been developed [4,16–18]. The proposed protocols consider node temperature as a primary metric for routing decisions. The main objective of the protocols is to maintain the temperature below some threshold and lower the temperature rise rate to avoid significant damage on human body tissue.

TARA [4] is one of the primitive protocols in this series. TARA forwards data packets based on localized temperature information and hop-count to the destination. It measures temperature considering heat generation due to radiation from antenna as well as power dissipation due to sensor circuitry. TARA maintains a neighbor table by exchanging neighborhood information, and forwarder node is selected based on the minimal temperature criteria. TARA estimates the temperature rise of its neighbors by listening to the neighbor activities and counting the number of packet transmission and reception. A hotspot is identified if the estimated temperature exceeds a certain threshold. TARA avoids the hotspot(s) by establishing an alternative route toward the destination using a withdrawal strategy where a packet is sent back to its previous sender if all the neighbors are identified as hotspots. The sender then attempts to select alternate route to detour the hotspot(s). After cooling the temperature beneath some threshold, those hotspots can be considered for later routing. Due to the withdrawal strategy, TARA suffers from high end-to-end delay, lower reliability, as well as high energy consumption since the packet needs to traverse many hops when it encounters a hotspot and will be detoured arbitrarily.

In order to address the problems of TARA, Bag and Bassiouni [16] proposed LTR. LTR also exploits greedy localized routing approach, where “coolest” neighbor is always chosen for data forwarding. To prevent a packet traversing unnecessarily with a large number of hops, a threshold parameter *MAX_HOPS* is defined, and if the hop-count of a packet exceeds the *MAX_HOPS*, then the packet is discarded. To avoid the routing loop, each packet maintains a small list of nodes which it has most recently visited and if the “coolest” neighbor is already in the list it is ignored to be as a forwarder and the second coolest neighbor is chosen. Because of the greedy approach, a packet in LTR is not always directed to the destination which significantly increases the hop count, thus resulting higher delay and lower reliability. In the same study, the authors presents ALTR, a variant of LTR, with an intent to minimize the packet delivery delay. In ALTR, a packet is forwarded similar to LTR until the hop-count of the packet reaches a threshold, *MAX_HOPS_ADAPTIVE*. However, a shortest path routing algorithm is used if the hop-count value exceeds the threshold. Although, compared to LTR, ALTR optimizes the end-to-end delay to some extent, but it wastes network bandwidth through unnecessary transmissions until the hop count reaches the threshold value. ALTR also allows a packet to traverse through hotspot(s) while utilizing the shortest hop routing.

HPR [18], as proposed by the same researchers of LTR and ALTR, follows an opposite mechanism as explained in ALTR. In HPR, a node usually forwards the packets using shortest hop routing. However, it chooses the “coolest” neighbor if it encounters a hotspot in the shortest hop-count path. Because of following the same routing strategy upon hop-count detection, HPR also behaves the same as LTR in terms of energy consumption, packet delivery delay and reliability.

LTRT [17] has been proposed to address the problems of LTR and ALTR. LTRT requires the knowledge of global network topology where a least temperature route is chosen from all possible routes from a sender node to a destination exploiting the Dijkstra's algorithm. LTRT, however, incurs much protocol overhead due to its dependency on end-to-end information. Furthermore, it only chooses the least temperature route which may not be the least delay path or reliable path.

QoS-aware routing protocols in the context of WBAN have not gained much attention so far. A QoS-aware routing framework for biomedical sensor network has been proposed by Liang *et al.* [11] that provides prioritized routing service and user specific QoS support exploiting cross-layer functionalities. This work considers user specific QoS requirements, wireless channel status, packet priority level, and sensor node's willingness to be a router in route selection. ZEQoS [12] presents an integrated energy and QoS-aware routing protocol following modular approach. It provides service differentiation through classifying traffic into three categories, and determines an end-to-end route that ensures the satisfying of QoS parameters based on respective traffic type. Exploiting the geographic location, DMQoS [13] has been proposed for biomedical wireless sensor networks. This protocol also provides differentiated service for four classes of traffic, and focuses on integrating energy-efficiency with QoS provisioning. All the existing QoS-aware protocols are mainly designed for inter-BAN communication, and ignore the thermal effects that could cause in the context of intra-BAN routing.

Lately, Monowar *et al.* [14] proposed a thermal-aware multi-constrained intra-body QoS-routing for WBAN that integrates the QoS provisioning issue with thermal-awareness in route selection for *in-vivo* sensor nodes. However, TMQoS is not well-suited for highly dynamic environment due to its dependency on global network information as we discussed in the previous section.

The lack of an efficient routing protocol integrating thermal-awareness with QoS provisioning for diverse traffic types thus motivates us to develop a localized routing solution for WBAN with *in-vivo* nodes that in one hand will address the highly dynamic nature of intra-BAN environment, and in other hand, will provide QoS-provisioning for heterogeneous traffic, minimizing the temperature rise along the network, also avoiding hotspot(s).

## 3. System Model and Preliminaries

### 3.1. System Model

**Figure 1:**
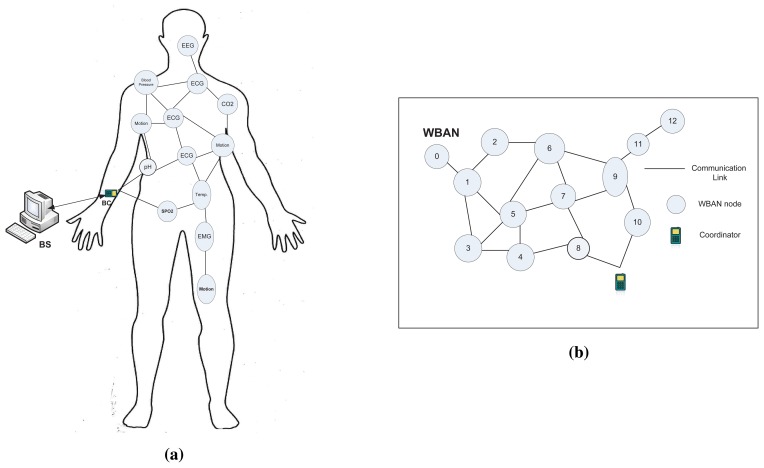
Network Model. (**a**) A Wireless Body Area Network (WBAN); (**b**) Communication Network Topology.

We consider a deployment scenario, in which diverse types of *in-vivo* nodes are placed in a human body forming a WBAN. A Body Coordinator(*BC*) is attached to the body surface, that serves as a central data sink for the WBAN as shown in Figure 1a. *In-vivo* nodes in a WBAN are usually energy constrained and responsible for sensing and transmission functions, while the *BC* could be equipped with external power supply having some advanced functionalities (*i.e.*, data aggregation, exchanging control and management packets *etc.*). The *BC* aggregates the data received data from the nodes, processes it, and then sends it to a Base Station (BS) or server through other networks (*i.e.*, cellular, WLAN or wired) as depicted in Figure 1a, and this communication paradigm is out of the scope of this paper.

The above deployment scenario can be modeled as a connectivity graph, **G** = (*N*,*E*), where *N* is the set of vertices representing the nodes in the network including *BC*, and *E* is the set of edges that represents the communication network topology as illustrated in Figure 1b. An edge, (*n_i_*, *n_j_*) ∈ *E*, *iff n_i_*,*n_j_* are within the communication range of each other. We assume every node *n_i_* uses fixed transmission power for the communication with neighboring nodes. We define the neighbor set of *n_i_*, denoted as *NB*(*n_i_*), are the nodes with which n*_i_* has direct edges. We consider all the communication links are symmetric, that is, if *n_i_* ∈ *NB*(*n_j_*), then *n_j_* ∈ *NB*(*n_i_*). To save energy and attain higher network connectivity, *in-vivo* nodes are assumed to have limited transmission range, thus data will be delivered to the BC through multiple hops. We assume that all the *in-vivo* nodes have forwarding capabilities (*i.e.*, forward other node's data) along with their sensing and transmission functions.

In this paper, we focus on designing a new localized and thermal-aware QoS routing protocol for a WBAN having *in-vivo* nodes. The term “localized routing” can be formally defined as

#### Definition 1.

*A routing protocol A is said to be a localized protocol, if given the information of a current node n_i_ and its neighbor set NB*(*n_i_*)*, the current node n_i_ can decide which neighboring node n_j_, n_j_* ∈ *NB*(*n_i_*) *is suitable to forward the data.*

To know the information of the neighboring nodes every node runs a HELLO protocol. For a localized routing to be effective, we assume that nodes are either stationary or having very low mobility, otherwise, nodes need to exchange HELLO packets more frequently which could be resource consuming. This assumption also agrees with the WBAN architecture with *in-vivo* nodes, since nodes are implanted inside the biological tissue of the human body. We also assume that in the network, node density is sufficient enough to prevent any “void” situation, where the void situation is termed as the situation between two neighboring nodes when there is no node in the network closer to one of them than the other.

### 3.2. Traffic Classification

One of the objectives of TLQoS is the QoS provisioning for diverse traffic types having different QoS requirements. Considering delay and reliability as the QoS metric, we classify the traffic as follows:

- *Critical(Cr)* traffic: This type of traffic has both the delay and reliability constraints. Examples include electroencephalogram (EEG), electrocardiogram (ECG) and Electromyography (EMG) *etc.* that generate real time continuous data which need to be delivered with a lower delay and higher reliability.
- *Delay constrained (Dc)* traffic: This type of traffic needs to be delivered with lower delay, however, it can tolerate some packet losses. Tele-medicine video streaming application traffic possesses such requirement.
- *Reliability constrained (Rc)* traffic: The traffic belong to this type requires higher reliability, without having any delay constraints. Example of this type includes respiration monitoring, pH-level monitoring *etc.* which can be processed offline, but packet losses for this type may cause severe consequences.
- *Regular (Rg)* traffic: Traffic of this type has no delay or reliability constraints. Traffic generated from a patient's regular vital sign monitoring applications such as temperature, pressure *etc.* corresponds to this class of traffic.

## 4. Design of TLQoS

### 4.1. Overview of TLQoS

**Figure 2:**
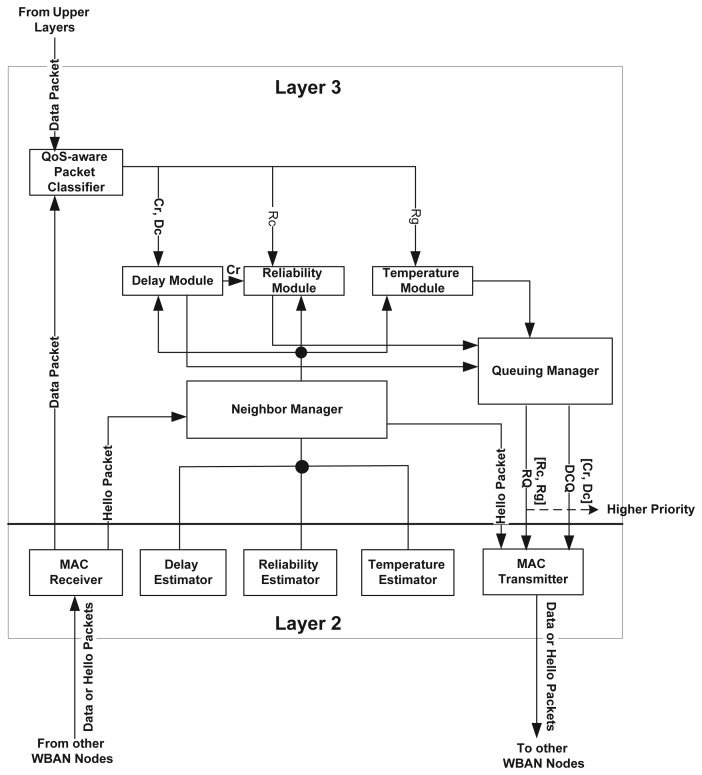
Protocol Architecture.

TLQoS is a thermal-aware localized routing protocol aiming to provision QoS for diverse traffic types based on their requirements. To meet the objective, the protocol is designed following a modular approach. Figure 2 illustrates the basic architecture of TLQoS. TLQoS exploits the cross layer interactions between layer-2 and layer-3.

Considering delay and reliability as the primary QoS constraints, a module is devoted to each of these constraints as depicted in Figure 2. The temperature module deals with the regular packet, (no delay and reliability constraints) and ensures that the packet reaches to the BC with a lower temperature route. The QoS-aware packet classifier classifies the packet according to their QoS demands and passes it to the respective module for further processing. The neighbor manager module runs the HELLO protocol that enables exchanging information among neighboring nodes, and maintains a neighbor table. This module interacts with delay estimator, reliability estimator and temperature estimator modules in layer-2 for acquiring the respective parameter values of a node. Upon exploiting the parameter values, the neighbor manager builds the HELLO packet. It also passes the neighbor table information to the delay module, reliability module and temperature module respectively to locally select the most appropriate neighboring node among the available candidates. The queuing manager module maintains two queues namely, Delay Constrained Queue (DCQ) and Regular Queue (RQ), and implements priority multi-queuing strategy that provides differentiated priority for diverse traffic types.

To implement the localized routing, TLoQoS adopts the potential based routing policy. The potential based routing is first proposed by Basu *et al.* [19] in the context of traditional network. However, this work defines an exclusive virtual potential field for each arbitrarily distributed destination that incurs huge overhead. Later on, Ren *et al.* proposed TADR [20] that exploits the potential field for localized routing decision in the context of Wireless Sensor Network (WSN) with a single sink. TADR mainly focuses on integrating congestion avoidance mechanism with routing functionalities. This work motivates us to adopt the potential based routing policy for QoS provisioning along with thermal-awareness in the context of WBAN having *in-vivo* nodes. In this policy, a node measures potentials for different parameters, and the trajectory of the packet is determined by the *force* from the potential fields. TLQoS defines four routing potential fields namely hop count potential, delay potential, reliability potential and temperature potential. The delay module, reliability module and temperature module respectively compute the related potential and forces for all the neighbors and choose the most suitable candidate that has the maximum force. While selecting the candidate node from the neighbors, all the modules avoid the hotspot node even if it has the maximum force. The process is repeated hop-by-hop until the packet reaches to the BC satisfying the requirements of desired QoS. Since, TLQoS employs greedy approach for choosing the next-hop node, and the QoS parameters as well as temperature parameter are time-variant, hence, routing loops might occur. However, integrating the QoS potential fields with time-invariant hop-count field, and controlling a configurable parameter can prevent the routing loops, and reduce the large number of hop traversal along the network. In the subsequent sections, we present how the potential fields and the relative forces are constructed, and then how the different modules compute the integrated potential and forces to decide a suitable loop-free route satisfying the respective QoS, also avoiding the hotspot.

### 4.2. Neighbor Manager

The neighbor manager module in TLQoS performs the following functions: (i) Executes the HELLO protocol; (ii) Builds and manages neighbor table; (iii) Interacts with the delay, reliability, and temperature estimator module to acquire the respective parameter values for the node; (iv) Provides the delay, reliability and temperature module with neighbor table information.

**Figure 3:**
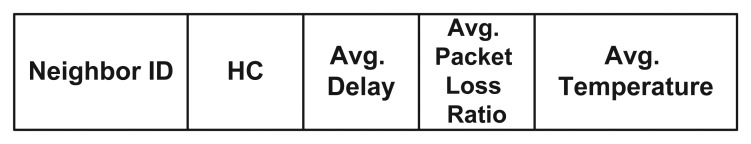
Neighbor Table Structure.

Periodically, or upon observing significant changes in the parameter values, a node broadcasts a HELLO packet to its neighbors. The HELLO packet includes node ID, hop-count to the BC, average node delay, average packet loss ratio, and average temperature of a node. This module maintains a neighbor table that assigns an entry for each neighboring node. The neighbor table structure is shown Figure 3. The table is updated upon each reception of the HELLO packet from a neighbor. The update includes addition of new entries when a new node enters into the node's neighborhood, deletion of a node entry if the update is not received for a particular time period, and updating information of the existing entries.

The frequency of HELLO packet exchange is also an important criteria to be considered. Since, most of the parameters (*i.e.*, delay, packet loss ratio, temperature) are highly dynamic, higher frequency of HELLO packet exchange would provide up-to-date information but would be resource consuming. However, lower frequency could result in stale information, also cause network connectivity loss. The HELLO packet interval selection mechanism is discussed later in this section.

### 4.3. Routing Potentials

TLQoS defines potential fields on the network over which diverse types of packets are routed to the BC. Let us consider a node, *n_i_* with potential, *P* generates a packet. To reach the *BC*, the packet needs to be forwarded to a neighbor *n_j_*, *n_j_* ∈ *NB*(*n_i_*). To choose a suitable “candidate” from *NB*(*n_i_*), we define a force acting on the packet at *n_i_*, as

(1) $$F(n_{i},n_{j})=\frac{P(n_{i})-P(n_{j})}{c_{n_{i},n_{j}}}$$

Here, *c_n_i__,_n_j__* denotes the link cost from *n_i_* to *n_j_*. Hence, the force is the potential difference between a node *n_i_* and each one of its neighbors. The packet is now directed to a neighbor *n_j_* ∈ NB(*n_i_*) for which the *F*(*n_i_*, *n_j_*) is maximum. In particular, a packet follows the direction of the steepest gradient to reach to the destination. To provide differentiated services, we assign four potential fields to a node *n_i_*. We define a set of potential fields to a node *n_i_* as $\text{P}\left(n_{i}\right)=\left[P_{n_{i}}^{h},P_{n_{i}}^{d},P_{n_{i}}^{r},P_{n_{i}}^{t}\right]$ . Here, $P_{n_{i}}^{h}$ , $P_{n_{i}}^{d}$ , $P_{n_{i}}^{r}$ , $P_{n_{i}}^{t}$ denote the hop-count potential, delay potential, reliability potential and temperature potential respectively.

#### 4.3.1. Hop-Count Potential Field

The hop-count potential field allows a packet to traverse to the *BC* following a shortest path. This field determines the node-depth from the *BC*. Let *p* be the packet at a node *n_i_*, and *HC*(*n_i_*) denotes the shortest path length in terms of hop-count from *n_i_* to the *BC*. Hence, the hop-count potential is defined as

(2) $$P_{n_{i}}^{h}=HC(n_{i})$$

Thus, the hop-count force that acts upon a neighbor *n_j_* ∈ *NB*(*n_i_*) from the node n*_i_* is given by

(3) $$F_{h}(n_{i},n_{j})=\frac{P_{n_{i}}^{h}-P_{n_{j}}^{h}}{c_{n_{i},n_{j}}}$$

Here, *c_n_i__,_n_j__* denotes the link cost from node *n_i_* to *n_j_*. Similar to [20], we consider the distance between two nodes as the link cost metric. The distance can be measured by several techniques such as measuring signal attenuation or received signal strength (RSSI) [21].

$P_{n_{i}}^{h}$ is time-invariant, and *F_h_*(*n_i_*, *n_j_*) allows a packet to traverse toward the *BC* with the least number of hops. Since, *NB*(*n_i_*) includes the nodes which are one-hop way from *n_i_*, so the hop-count difference will be 0, −1 or 1. Thus, the hop-count force *F_h_*(*n_i_*, *n_j_*) will have either of the following values: 0, $\frac{-1}{c_{n_{i},n_{j}}}$ , $\frac{1}{c_{n_{i},n_{j}}}$ .

#### 4.3.2. Delay Potential Field

We define the delay potential of a node *n_i_* as

(4) $$P_{n_{i}}^{d}=D(n_{i})$$

Here, the function *D*(*n_i_*) refers to the normalized node delay at *n_i_* and defined by

(5) $$D(n_{i})=\frac{D_{n_{i}}^{avg}}{D_{p}^{req}}$$

Here, $D_{n_{i}}^{avg}$ denotes the average node delay at *n_i_*, and $D_{p}^{req}$ is the end-to-end delay requirement of a packet, in other words, the packet deadline. Thus, delay is normalized to the packet delay requirement.

Then, we define the delay force, *F_d_*(*n_i_*,*n_j_*) from node *n_i_* to *n_j_* ∈ *NB*(*n_i_*) as

(6) $$F_{d}(n_{i},n_{j})=\frac{P_{n_{i}}^{d}-P_{n_{j}}^{d}}{c_{n_{i},n_{j}}}$$

*F_d_*(*n_i_*, *n_j_*) allows a packet to traverse through the nodes with minimum latency on its path to the *BC*. The delay potential field, however, is time variant which changes dynamically, and thus is not loop free. However, it can be avoided by integrating the field with time-invariant hop-count potential field which will be described later in this section.

#### 4.3.3. Reliability Potential Field

The reliability potential field, $P_{n_{i}}^{r}$ on a node *n_i_* is defined as

(7) $$P_{n_{i}}^{r}=R(n_{i})$$

Here, *R*(*n_i_*) denotes the average packet loss ratio at node *n_i_*. Hence, the lower the ratio, the higher the reliability of the node.

The reliability force, *F_r_*(*n_i_*, *n_j_*) acting on node *n_i_* to *n_j_* ∈ *NB*(*n_i_*) is given by

(8) $$F_{r}(n_{i},n_{j})=\frac{P_{n_{i}}^{r}-P_{n_{j}}^{r}}{c_{n_{i},n_{j}}}$$

Thus, driven by this potential, a packet moves along the nodes having least packet loss ratio, *i.e.*, reliable nodes on its path to the *BC*. This potential is also time-variant and is not loop free. However, combining with hop-count potential would result a loop free path as will be discussed later.

#### 4.3.4. Temperature Potential Field

The temperature potential field is defined as

(9) $$P_{n_{i}}^{t}=T(n_{i})$$

Here, *T*(*n_i_*) denotes the normalized temperature at node *n_i_* which is defined as

(10) $$T(n_{i})=\frac{T_{n_{i}}^{avg}}{T^{thr}}$$

Here, $T_{n_{i}}^{avg}$ refers to the average temperature at node *n_i_*, and *T^thr^* denotes the temperature threshold for hotspot detection. If the value of $T_{n_{i}}^{avg}$ exceeds *T^thr^*, then *T^thr^* will be assigned to $T_{n_{i}}^{avg}$ .

Then, we define the temperature potential force from a node *n_i_* to *n_j_*∈ *NB*(*n_i_*) as

(11) $$F_{t}(n_{i},n_{j})=\frac{P_{n_{i}}^{t}-P_{n_{j}}^{t}}{c_{n_{i},n_{j}}}$$

This force drives a packet to traverse along the nodes having least temperature on its path to the *BC.* Similar to delay and reliability potential, it is also time-variant that might change dynamically, also the routing loop issue is addressed the similar way to delay and reliability potential field as to be discussed later.

### 4.4. Hybrid Potential and Routing Loop Avoidance

Among the four potential fields we defined, only the hop-count potential is time-invariant, and the remaining potentials change dynamically. In [19], the authors proved that, a potential based routing with time invariant potential is loop-free. Thus, exploiting the delay, reliability and temperature potentials independently in routing decisions would result in routing loops. In TLQoS, we define the hybrid potential for each of the time-variant potential field to address this situation. Hybrid potential field combines a time-invariant potential (*i.e.*, hop-count in TLQoS) with a time-variant potential field, and together impacts on the routing decision for particular traffic type. Different combination patterns are possible, however, for simplicity and tractability, we adopt linear combination of the potential fields.

Let $P_{n_{i}}^{Hd}$ , $P_{n_{i}}^{Hr}$ , and $P_{n_{i}}^{Ht}$ denote the hybrid potential field for delay, reliability and temperature potential respectively. Thus, the hybrid potentials at node *n_i_* are defined as

(12) $$P_{n_{i}}^{Hd}=\left(1-\gamma \right)P_{n_{i}}^{h}+\gamma P_{n_{i}}^{d}$$

(13) $$P_{n_{i}}^{Hr}=\left(1-\gamma \right)P_{n_{i}}^{h}+\gamma P_{n_{i}}^{r}$$

(14) $$P_{n_{i}}^{Ht}=\left(1-\gamma \right)P_{n_{i}}^{h}+\gamma P_{n_{i}}^{t}$$

Here, *γ* is an adjustable parameter where, 0 ≤ *γ* ≤ 1. In TLQoS, *γ* has significant influences on routing decision since controlling this value decides which part of the hybrid potential field will dominate.

Based on the hybrid potential field, we also define the hybrid forces from node *n_i_* to *n_j_* ∈ *NB*(*n_i_*):

(15) $$F_{Hd}(n_{i},n_{j})=\frac{P_{n_{i}}^{Hd}-P_{n_{j}}^{Hd}}{c_{n_{i},n_{j}}}$$

(16) $$F_{Hr}(n_{i},n_{j})=\frac{P_{n_{i}}^{Hr}-P_{n_{j}}^{Hr}}{c_{n_{i},n_{j}}}$$

(17) $$F_{Ht}(n_{i},n_{j})=\frac{P_{n_{i}}^{Ht}-P_{n_{j}}^{Ht}}{c_{n_{i},n_{j}}}$$

The above equations can be rewritten as

(18) $$F_{Hd}(n_{i},n_{j})=(1-\gamma )F_{h}(n_{i},n_{j})+\gamma F_{d}(n_{i},n_{j})$$

(19) $$F_{Hr}(n_{i},n_{j})=(1-\gamma )F_{h}(n_{i},n_{j})+\gamma F_{r}(n_{i},n_{j})$$

(20) $$F_{Ht}(n_{i},n_{j})=(1-\gamma )F_{h}(n_{i},n_{j})+\gamma F_{t}(n_{i},n_{j})$$

In TLQoS, a packet *p* with a particular traffic type is forwarded to a neighbor for which the relevant force is maximum, particularly, the neighbor in the direction of the steepest gradient for that related force. TLQoS purposefully controls the value of *γ* which changes dynamically depending on the number of hops a packet already traversed. This value significantly influences in routing decision. In particular, assigning more weights with time-variant potential field part dominates that potential in routing decision while more weights with time-invariant hop-count potential drives a packet to traverse over a shortest path to the *BC*. While time-variant potential dominates, routing loop might be created as the packet can be forwarded to a neighbor that is in the same depth (*i.e.*, same hop-count) or even it can be forwarded backward to a neighbor with higher hop-count. This situation prevails only when the hybrid potential of all the lower-depth neighbors is greater than the hybrid potential of the node itself.

In TLQoS, for any node, the related potential can increase by *γ* at maximum. This is the situation when the $D_{n_{i}}^{avg}=D_{p}^{req}$ , or the average packet loss ratio, *R*(*n_i_*) = 1, or $T_{n_{i}}^{avg}=T^{thr}$ . Thus, a node will not choose a neighbor with lower hop-count if and only if *γ* > 1 − *γ*. Hence, we get, $\gamma >\frac{1}{2}$ . TLQoS initialize the value of *γ* with some value greater than $\frac{1}{2}$ , allowing a packet to be traversed to a neighbor on which the related time-variant potential will be dominating and any neighbor can be chosen irrespective of its hop-count value. However, to avoid the routing loops or avoiding a packet roaming unnecessarily around the network we impose two conditions:

- *Condition 1:* A node will not choose a neighbor from which it just received a packet.
- *Condition 2:* Whenever the hop-count value of a packet exceeds a maximum hop-count threshold, *HC^max^*, the parameter *γ* is changed as *γ* = *γ* − 0.1 for every hop the packet traverses until it reaches to the *BC.*

Condition 1 prevents a packet to be exchanged between two nodes back and forth, thus avoids routing loops. Condition 2 avoids a packet moving around a large number of hops, also prevents routing loops. With the decreasing value of *γ*, the time-invariant potential will be dominating that drives the packet to be closer to the *BC* following the shortest path route.

### 4.5. Delay Module

The delay module aims for choosing a potential forwarder for delay-constrained and critical traffic type. Algorithm 1 presents route selection procedure used by delay module.


**Algorithm 1** Route selection algorithm at delay module
**INPUT** (a packet *p* ∈ *Cr, Dc*, NT)1.**if**
*p.HC* > *HC^max^* {Condition-2} **then**2. *γ*=*p*.*γ*3. *γ* = *γ* − 0.14. *p*.*γ*=*γ*5.**else**6. *γ* =*p*.*γ*7.**end if**8.**for all**
*n_j_* in NT **do**9. **if**
$T_{n_{i}}^{avg}<T^{thr}$ {Hotspot Avoidance} **then**10.  **if**
*n_j_* ∉ *RS* {Condition-1} **then**11.   *c_n_i_,n_j__* = *dst_n_i_,n_j__*12.   Calculate 
$P_{n_{i}}^{d}$ and 
$P_{n_{j}}^{d}$ according to Equations (4) and (5)13.   Calculate 
$P_{n_{j}}^{Hd}$ according to Equation (12)14.   Calculate *F_h_*(*n_i_*,*n_j_*) according to Equation (3)15.   Calculate *F_d_*(*n_i_*,*n_j_*) according to Equation (6)16.   Calculate *F_Hd_*(*n_i_*,*n_j_*) according to Equation (18)17.  **end if**18. **end if**19.**end for**20.Select *n_j_* with MAX-*F_Hd_*(*n_i_*, *n_j_*), 
$\text{MIN}-P_{n_{j}}^{Hd}$, 
$\text{MIN}-P_{n_{j}}^{h}$, Random in turn21.**if** p.PacketType=Cr **then**22. Call Reliability Module with *p* ∈ *Cr* as parameter23.**else**24. Call Queuing Manager with *p* ∈ *Dc* as parameter25.**end if**


The algorithm first checks the condition-2 for the delay sensitive packets. The recent value of *γ* is extracted from the packet header. If the hop-count value of the packet exceeds maximum hop-count threshold, it resets the value of *γ* (line-2,3). The current value of *γ* is also updated in the packet (line-4). Otherwise, the *γ* will not change. The delay module avoids the hotspot by ignoring the neighbors for which the average temperature exceeds some hotspot threshold, *T^thr^* (line-9). Condition-1 is applied in line-10 to avoid the routing loop with recent sender, *RS.* For the remaining neighbors other than the hotspot(s) and recent sender, the algorithm measures the required potentials and forces (line 11–16). Finally, it chooses the neighbor with maximum hybrid delay force as its potential forwarder. However, if more than one neighbor possesses the same maximum force, then the potential forwarder will be selected based on minimum hybrid delay potential, minimum hop-count potential successively. If the forwarder can not be determined even after this, the algorithm chooses it randomly.

After selecting the suitable forwarder node, the algorithm checks the packet type. For a *critical* packet, reliability module is called for further forwarder selection that ensures the packet reliability. However, if the packet type is delay constrained, the queuing manager module will be called to place the packet in a proper queue for transmission.

### 4.6. Reliability Module

The reliability module chooses a forwarder locally that ensures the higher reliability for *Rc* and *Cr* traffic type. Algorithm 2 shows the route selection procedure at this module.


**Algorithm 2** Route selection algorithm at reliability module
**INPUT** (a packet *p* ∈ *Cr, Rc*, NT)1.**if**
*p.HC* > *HC^max^* {Condition-2} **then**2. *γ*=*p*.*γ*3. *γ* = *γ* − 0.14. *p*.*γ*=*γ*5.**else**6. *γ* =*p*.*γ*7.**end if**8.**for all**
*n_j_* in NT **do**9. **if**
$T_{n_{j}}^{avg}<T^{thr}$ {Hotspot Avoidance} **then**10.  **if**
*n_j_* ∉ *RS* {Condition-1} **then**11.   *c_n_i__,_n_j__* = *dst_n_i__,_n_j__*12.   Calculate 
$P_{n_{i}}^{r}$ and 
$P_{n_{j}}^{r}$ according to Equation (7)13.   Calculate 
$P_{n_{j}}^{Hr}$ according to Equation (13)14.   Calculate *F_h_*(*n_i_, n_j_*) according to Equation (3)15.   Calculate *F_r_*(*n_i_, n_j_*) according to Equation (8)16.   Calculate *F_Hr_*(*n_i_, n_j_*) according to Equation (19)17.  **end if**18. **end if**19.**end for**20.Select *n_j_* with MAX-*F_Hr_*(*n_i_, n_j_*), 
$\text{MIN}‐P_{n_{j}}^{Hr}$, Random in turn21.Call Queuing Manager with *p* ∈ *Cr* or *Rc* as parameter


The algorithm follows a similar procedure to the delay module in applying Condition-1, Condition-2 and Hotspot avoidance mechanism. Since reliability is the concerned metric here, the algorithm measures the related reliability potential and forces as shown in line (12–16). The algorithm chooses the potential forwarder that has the maximum hybrid reliability force, and takes into account a neighbor with minimum hybrid reliability potential if more than one neighbor has the same maximum value of *F_Hr_*(*n_i_, n_j_*)*.* Unlike the delay module, we ignore the minimum hop-count potential value as an option, as delay is not the concerned metric in this case. The algorithm goes for random choice if none of the mentioned condition works. After determining an appropriate forwarder node ensuring the reliability, the algorithm calls the queuing manager module to place the *Cr* or *Rc* packet in the relevant queue.

### 4.7. Temperature Module

This module focuses on route selection for regular packet type. Since the *Rg* traffic has no QoS constraints, this module chooses a forwarder with lower temperature among the neighbors. The route selection procedure used in this module is depicted in Algorithm 3.


**Algorithm 3** Route selection algorithm at temperature module
**INPUT** (a packet *p* ∈ *Rg*, NT)1.**if**
*p.HC* > *HC^max^* {Condition-2} **then**2. *γ*=.*pγ*3. *γ* = *γ* − 0.14. *p*.*γ*=*γ*5.**else**6. *γ* =*p*.*γ*7.**end if**8.**for all**
*n_j_* in NT **do**9. **if**
$T_{n_{j}}^{avg}<T^{thr}$ {Hotspot Avoidance} **then**10.   **if**
*n_j_* ∉ *RS* {Condition-1} then11.   *c_n_i__,_n_j__* = *dst_n_i__,_n_j__*12.   Calculate 
$P_{n_{i}}^{t}$ and 
$P_{n_{j}}^{t}$ according to Equations (9) and (10)13.   Calculate 
$P_{n_{j}}^{Ht}$ according to Equation (14)14.   Calculate *F_h_*(*n_i_, n_j_*) according to Equation (3)15.   Calculate *F_t_*(*n_i_, n_j_*) according to Equation (11)16.   Calculate *F_Ht_*(*n_i_, n_j_*) according to Equation (20)17.  **end if**18. **end if**19.**end for**20.Select *n_j_* with MAX-*F_Ht_*(*n_i_*, *n_j_*), 
$\text{MIN}‐P_{n_{j}}^{Ht}$, Random in turn21.Call Queuing Manager with *p* ∈ *Rg* as parameter


Similar to the other module, temperature module also executes the condition-1, condition-2 and hotspot avoidance mechanism following the same procedure. The difference lies in measuring the parameter values that considers temperature related potential and forces as depicted in line (12–16) of the algorithm. The potential forwarder is chosen having the maximum hybrid temperature force. If more than one neighbor has the same maximum value of hybrid potential force, minimum hybrid temperature potential is used to select the potential forwarder. If the potential forwarded is still not determined, it will be chosen randomly. At the end, queuing manager will be called with *Rg* packet as a parameter.

### 4.8. Queuing Manager

The queuing manager in TLQoS exploits a multi-queue priority policy where higher priority is given to the delay constrained traffic (*Cr* and *Dc*) than the non-delay constrained (*Rc* and *Rg*) traffic types. To achieve this, this module maintains two separate queues, namely DCQ and RQ for storing delay constrained packets, and non-delay constrained packets respectively The DCQ has the higher priority over RQ. It implies that the packets from RQ will not be sent until the DCQ becomes empty. However, it might cause starvation problem where the lower priority traffic could be indefinitely blocked by higher priority traffic. To avoid the problem we adopt the similar mechanism as described in [13], in which a time-out based policy have been employed. In this policy, a packet will be moved to a higher priority queue (irrespective of its type) if it waits in a lower priority queue until a time-out occurs. The multi-queue priority policy addresses the priority contention between packets in the same node. However, to address the priority contention between neighboring nodes, the MAC protocol could be modified which is beyond the scope of the paper.

### 4.9. Parameter Estimation

#### 4.9.1. Hop-Count Measurement

The hop-count parameter is significant in TLQoS as it is the only time-invariant parameter considering the low-mobility or lower frequency in topology change. The neighbor manager sets the hop-count of a node upon receiving HELLO packet from its neighbors. Initially, all nodes except the sink will initialize their hop-count value to a very large number while the hop-count for sink will be 0. Upon receiving HELLO packet from a neighbor, *n_j_* ∈ *NB*(*n_i_*); a node, *n_i_* updates its hop-count value as follows:

- IF (*HC*(*n_j_*) + 1 ≤ *HC*(*n_i_*)), then *HC*(*n_i_*) = *HC*(*n_j_*) + 1

Thus, the hop-count value is determined as the minimum hop-count to the *BC i.e.*, the shortest path length. However, if a node *n_i_* detects any topological changes then it recalculates the hop-count as

(21) $$HC(n_{i})=\min[HC(n_{j})]+1,\forall n_{j}\in NT$$

#### 4.9.2. Delay Estimation

The Delay Estimator module of a node *n_i_* estimates the average node delay, $D_{n_{i}}^{avg}$ as follows:

(22) $$D_{n_{i}}^{avg}=\bar{D_{n_{i}}^{DCQ}}+\bar{D^{tr}}+D_{n_{i}}^{proc}+D^{prop}$$

Here, $\bar{D_{n_{i}}^{DCQ}}$ denotes the average queuing delay of DCQ at node *n_i_*, $\bar{D^{tr}}$ is the average transmission delay, $D_{n_{i}}^{proc}$ is the processing delay at node *n_i_* , and *D^prop^* is the propagation delay. Among these four types, $\bar{D_{n_{i}}^{DCQ}}$ and $\bar{D^{tr}}$ dominates the total latency. $D_{n_{i}}^{proc}$ is trivial and assumed to be same for all packets and *D^prop^* is the light speed propagation delay, hence these delays are ignored.

In node delay estimation, TLQoS considers only delay sensitive packets that include Cr and Dc traffic, since the delay parameter is considered for the route selection of delay sensitive packets only as discussed in Section 4.5.

Queuing delay for DCQ, $D_{n_{i}}^{DCQ}$ is usually the interval between the time when a delay sensitive packet enters into the queue and the time it is ready for transmission. The running average of queuing delay, $\bar{D_{n_{i}}^{DCQ}}$ is estimated using the Exponentially Weighted Moving Average (EWMA) formula [22] as follows:

(23) $$\bar{D_{n_{i}}^{DCQ}}=(1-\beta )\bar{D_{n_{i}}^{DCQ}}+\beta D_{n_{i}}^{DCQ}(curr)$$

Here, $D_{n_{i}}^{DCQ}\left(curr\right)$ is the current observation of queuing delay at *n_i_, β* is a weighting factor which satisfies 0 < *β* ≤ 1. The value of *β* can be empirically chosen. We use the value *β* = 0.2 in our simulation.

The transmission delay, *D^tr^* is defined as the duration from the time a packet begins to be served by the MAC layer to the time when the acknowledgment of the packet is received. The transmission delay is also interpreted as service time of the MAC layer. Let *t*_0_ be time when a packet arrives at the head of the queue and ready for transmission, *t_ack_* is the time when the acknowledgment of packet is received, *L_ack_* is the length of acknowledgment packet and *bw* is the bandwidth. Hence, *D^tr^* is estimated as

(24) $$D^{tr}=t_{ack}-\frac{L_{ack}}{bw}-t_{0}$$

The running average of transmission delay, $\bar{D^{tr}}$ is estimated used EWMA formula as follows

(25) $$\bar{D^{tr}}=(1-\beta )\bar{D^{tr}}+\beta D^{tr}(curr)$$

Here, *D^tr^*(*curr*) denotes the current observation of transmission delay and the *β* is similar to Equation (23).

#### 4.9.3. Reliability Estimation

TLQoS estimates the reliability of a node in terms of its packet loss rate. Thus, the less is the packet loss rate, the more is the reliability. The reliability estimator module measures the average packet loss ratio at a node *n_i_*. Let *f* be the sum of packet losses over a time window, *δt* sent by node, *n_i_*, and *r* be the number of successfully acknowledged packets over that time window. Hence, the mean $\bar{\mu }=\frac{f}{r+f}$ represents the packet loss ratio of node *n_i_* for that time window. This per window packet loss rate is then averaged with the previous measurements using Window Mean with EWMA (WMEWMA) formula [23] as follows:

(26) $$R(n_{i})=R(n_{i})\times \alpha +(1-\alpha )\times \bar{\mu }$$

Here, *α* is a smoothing factor which controls the history of the estimator and 0 < *α <* 1. The value of *α* is also empirically chosen and we use the value 0.4 in our simulation.

#### 4.9.4. Temperature Estimation

TLQoS exploits the temperature estimation procedure as described in [3,4]. In particular, the temperature rise of the tissue surrounding the WBAN nodes is measured. The major sources that are predominant for thermal increase of the implant devices include radiation from the node antenna and power dissipation of the implanted electronics. Specific absorption rate (SAR) is used to determine the level of radiation being absorbed by body tissue. The space near the antenna is known as near field, the extent of which is $\frac{\lambda }{2\pi }$ , where λ is the radio frequency (RF) wavelength for the wireless communication. According to [10,24], the SAR in the near field ( $\leq \frac{\lambda }{2\pi }$ ) and far field ( $>\frac{\lambda }{2\pi }$ ) can be estimated as follows:

(27) $$SAR_{NF}=\frac{\sigma \mu \omega }{\rho \sqrt{\sigma^{2}+{ɛ}^{2}\omega^{2}}}{\left(\frac{Idl\text{sin}\theta e^{-\alpha R}}{4\pi }\left(\frac{1}{R^{2}}+\frac{\gamma }{R}\right)\right)}^{2}$$

(28) $$SAR_{FF}=\frac{\sigma }{\rho }{\left(\frac{{\bar{\alpha }}^{2}+{\bar{\beta }}^{2}}{\sqrt{\sigma^{2}+\omega^{2}{ɛ}^{2}}}\frac{Idl}{4\pi }\right)}^{2}\frac{{\text{sin}}^{2}\theta e^{-2\alpha R}}{R^{2}}$$

where, *R* is the distance from the source to the observation point, *γ* is the propagation constant, *dl* is the length of short conducting wire for a short dipole antenna, *σ* is the medium conductivity, *I* is the amount of current, *ϵ* is the relative permittivity, *µ* is the permeability, *ρ* is the mass density and *sinθ* = 1.

The power dissipation of the sensor node circuitry, can be quantified as power dissipation density, *P_c_* (the power consumed by the sensor circuitry divided by the volume of the sensor) [3]. Considering both the sources for temperature rise, the temperature of a node at a grid point (x,y) at time *t*, denoted as *T^t^*(*x, y*) can be estimated as follows [3]:

(29) $$\begin{array}{ll} T^{t}(x,y) & =\left(1-\frac{\Delta_{t}b}{\rho C_{p}}-\frac{4\Delta_{t}K}{\rho C_{p}\Delta^{2}}\right)T^{t-1}(x,y)+\frac{\Delta_{t}}{C_{p}}SAR\\ & +\frac{\Delta_{t}b}{\rho C_{p}}T_{b}+\frac{\Delta }{\rho C_{p}}P_{c}+\frac{\Delta_{t}K}{\rho C_{p}\Delta^{2}}(T^{t-1}(x+1,y)\\ & +T^{t-1}(x,y+1)+T^{t-1}(x-1,y)+T^{t-1}(x,y-1)) \end{array}$$

The Equation (29) exploits the Finite-Difference Time-Domain (FDTD) modeling technique [25] that discretizes the differential form of time and space, and the problem space is discretized into small grids. In Equation (29), Δ*_t_* is the discretized time step, Δ is the discretized space step, *b* is the blood pressure perfusion constant, *C_p_* is the specific heat of the tissue, Tb is the fixed blood temperature, *K* is the thermal conductivity of the tissue. From Equation (29), the temperature of a node at grid point (x, y) at time *t* can be determined through a function of the temperature at (x, y) at time *t* − 1, and the function of the temperature of neighboring nodes at grid points ((x + 1, y), (x, y + 1), (x − 1, y) and (x, y − 1);. Since the sensor nodes are surgically implanted, hence the position of the nodes are fixed and can be easily known. Once the properties of the tissue, the properties of blood flow, and the heat absorbed by the tissue will be obtained, the temperature at a given time can be measured. Reference [3] provides the details of this temperature estimation modeling.

## 4.10. HELLO Packet Interval

The HELLO packet interval should be carefully chosen to reflect the actual network status in a dynamic wireless environment and maintain the network connectivity. In addition, the interval should not be too short to cause unnecessary resource consumption of the resource-constrained WBAN. Similar to the mechanism as discussed in [20], TLQoS exploits two HELLO packet interval, namely minimum HELLO interval denoted as *HI^min^* and maximum HELLO interval denoted as *HI^max^.* Instead of sending the updates for every changes in the parameter, *HI^min^* is used for periodic updates reflecting the time variant parameters such as delay, reliability and temperature. On the other hand, *HI^max^* is chosen as higher value that ensures the network connectivity reflecting a time-invariant parameter such as hop-count. We define three threshold for HELLO packet update considering three time-variant parameters, namely delay update threshold, $D_{up}^{th}$ ; reliability update threshold, $R_{up}^{th}$ ; and temperature update threshold, $T_{up}^{th}$. TLQoS triggers a HELLO packet if any of the following events occur:

- *Event-1* The recent change in delay, reliability and temperature exceeds $D_{up}^{th}$ or $R_{up}^{th}$ or $T_{up}^{th}$ since the last update message sent at *HI^min^* and current value of *HI^min^* expires
- *Event-2* The hop-count of a node changes due to changes in topology
- *Event-3* The current value of *HI^max^* expires since the last HELLO packet sent at *HI^max^*

Event-1 ensures that if no significant changes occur in delay, reliability or temperature since the last HELLO packet interval sent at *HI^min^*, the HELLO packet will not be transmitted. Event-2 ensures the requirement of taking immediate action due to the time-invariant parameter hop-count changes. Event-3 is used to maintain the network connectivity no matter whether any parameter changes or not.

## 5. Performance Evaluation

In this section, we evaluate the performance of TLQoS based on simulation.

### 5.1. Simulation Environment

We consider a network area of 10*m* × 10*m*, in which 50 nodes are deployed in uniform random distribution, and the *BC* is placed in the center of the network. In the simulation, nodes send data to the *BC* through multi-hop communication. The simulation program has been developed in C++.

Table 1 describes the simulation parameters. We obtain the values for tissue properties and dielectric characteristics from [26,27]. Initially, the temperature of all the sensors are set to 37 ^°^C. Among the 50 implant nodes, traffic classes are distributed as follows: 5 nodes are assigned *Cr* traffic, 10 nodes are assigned *Dc* traffic, *Rc* traffic is assigned to 15 nodes and the remaining 20 nodes are assigned to *Rg* traffic. Nodes are chosen randomly during the distribution. We implement the basic functionalities of non-beacon enabled mode of IEEE 802.15.4 MAC protocol with the default values [28]. As benchmark protocols, we choose TARA, ALTR and TMQoS to compare with TLQoS. TARA and ALTR are chosen as both the protocols employ hop-by-hop approach in making routing decision, while TMQoS is the only protocol that provides QoS provisioning with end-to-end route maintenance. Each simulation has been performed for 1000 s and we averaged the value obtained over 10 random runs.

### 5.2. Performance Metrics

We used the following metrics to evaluate the performance of TLQoS.

*Average End-to-End Latency*. End-to-End latency of a packet is measured as the time difference between the packet generation time and the time when it is received by the BC. Delays experienced by distinct delay-sensitive data packets (Cr and Dc) are averaged over the total number of delay-sensitive packets received by the BC.
*On time Packet delivery ratio*. It is the ratio of the total number of delay-sensitive packets received by the BC within the deadline to the total number of delay sensitive packets generated by the WBAN nodes.
*Reliability*. It is the ratio of the total number of unique reliability-sensitive packets (Cr and Rc) received by the BC to the total number of reliability-sensitive packets generated by the WBAN nodes.
*Average Temperature Rise*. The average temperature rise of the nodes presents the average change in temperature of the nodes from that at the initial simulation period.
*Average Energy Consumption*. This parameter considers the average energy consumption of the nodes due to transmission and reception of packets. In our simulation, each packet transmission by a node consumes 0.2 units of energy and each reception consumes 0.1 units of energy.

### 5.3. Simulation Results

We first evaluate the performance of TLQoS considering the impacts of traffic load and bit error rates (BER). We further investigate the impact of delay deadline on QoS aware protocols such as TMQoS and TLQoS for the delay sensitive packets. The results are discussed in the following subsections.

#### 5.3.1. Impact of Traffic Load

**Figure 4:**
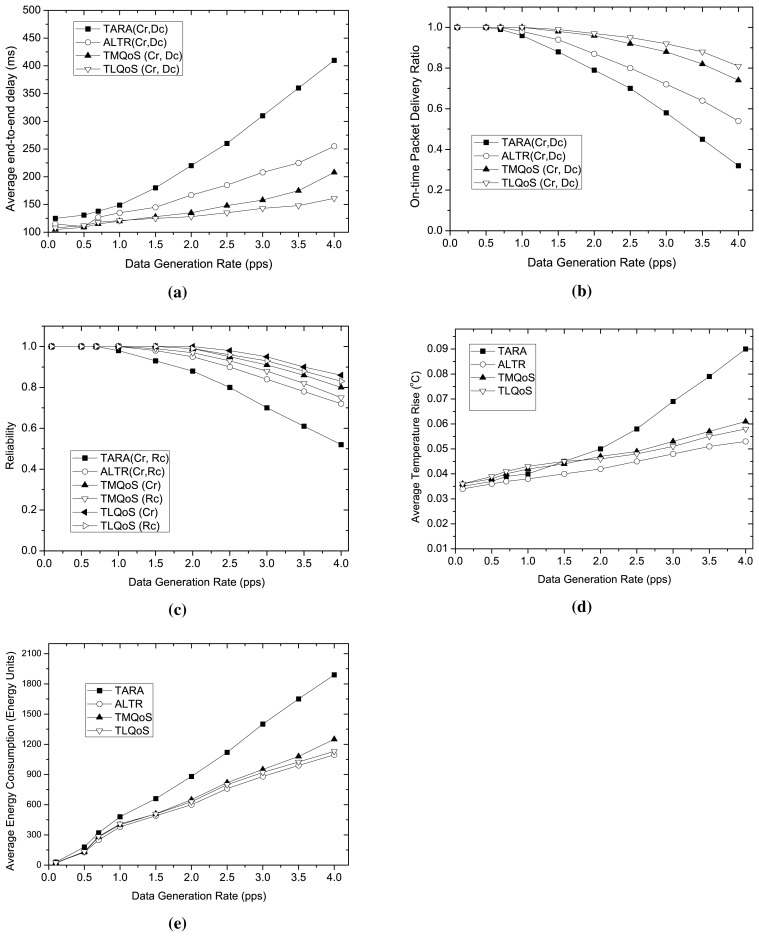
Performance Comparison for different traffic loads. (**a**) Average End-to-End delay varying data generation rate; (**b**) On Time Packet Packet Delivery Ratio varying data generation rate; (**c**) Reliability varying data generation rate; (**d**) Average Temperature Rise varying data generation rate; (**e**) Average Energy Consumption varying data generation rate.

Figure 4 illustrates the performance of the protocols varying traffic load. In this study, we vary the traffic load by varying data generation rate in terms of packets generated per second. The bit error rate varies randomly ranging from 10^−6^ to 10^−2^. Here, the impact of traffic load is investigated on the performance metrics for the related traffic classes. Although TARA and ALTR do not support traffic differentiation, however, the data related to the corresponding traffic class was collected exploiting the node-ID that generates traffic of a particular type.

We evaluate the average end-to-end delay only for delay sensitive packets. As the traffic load increases, the average end-to-end delay increases for all the protocols as depicted in Figure 4a. TARA exhibits the worst performance in high traffic load due to its withdrawal strategy while a hotspot is encountered. Compared to TARA, ALTR shows better performance in this regard, as it avoids unnecessary hop traversal applying the shortest path algorithm for packet routing after a hop-count threshold is reached. Considering delay parameter in route selection, both TMQoS and TLQoS achieve the significantly lower end-to-end delay compared to the non-QoS aware protocols. During low traffic load, TLQoS and TMQoS show almost similar performance in end-to-end delay, however, TLQoS excels the delay performance than TMQoS in high traffic load. The reason behind is that, TMQoS adopts end-to-end approach in taking routing decision which delays the propagation of routing information from BC to the source at high traffic load due to the increased contention, in other words, the convergence time is prolonged resulting stale information during high traffic load. In contrast, TLQoS employs local selection for choosing the least delay node from its neighbors. Despite the greedy approach, TLQoS avoids the unnecessary packet transmission along the network, and tends to follow less hop-count path to the *BC* due to hybrid potential field and routing loop avoidance mechanism.

The impact of traffic load on on-time delivery ratio is depicted in Figure 4b. This metric considers the effective reliability for delay sensitive packets, in particular, it takes into account the number of successful delivery of delay sensitive packets within the specified deadline. Considering 300 ms as a deadline for delay sensitive packets, TLQoS demonstrates the best performance regarding on-time delivery ratio among all the protocols because of its localized QoS provisioning approach. Due to the high latency at high traffic load, and higher packet losses, TMQoS shows poor performance in on-time delivery ratio compared to TLQoS. TARA, because of its poor delay performance even in moderate traffic load, the on-time delivery ratio drops significantly as the traffic load increases. The poor reliability performance also influences this result as to be discussed later. ALTR, however, achieves much higher on-time delivery ratio than TARA in all traffic loads. Still, this performance does not exceed the performance of QoS-aware protocols like TMQoS and TLQoS due to the lack of exploiting QoS parameters as routing metric.

The reliability performance for reliability sensitive packets (Cr and Rc) varying traffic load is shown in Figure 4c. With the increasing traffic load, the reliability drops for all the protocols. This is because, at high traffic load, the media contention and node congestion increases, resulting higher packet drops. Because of the excessive packet transmission, the reliability of TARA drops sharply as the traffic load increases. Comparatively, ALTR shows better performance than TARA as it avoids excessive packet transmission because of employing the shortest path routing. The QoS-aware protocols outperform both TARA and ALTR considering reliability as one of the routing metrics for reliability sensitive packets. Both TMQoS and TLQoS treat the *Cr* and *Rc* packet separately in taking their routing decision. Because of employing both path reliability and data reliability, *Cr* packet achieves higher reliability than *Rc* for both the protocols. Overall, the reliability performance of TLQoS is better than TMQoS for both the packet types, as during high traffic load, TLQoS adapts the dynamic changes in network status much more efficiently than TMQoS because of its localized approach in routing decision.

We evaluate the average temperature rise for different traffic loads as shown in Figure 4d. ALTR shows the best performance in this regard as it route all types of packet through the “cooler” node and avoids unnecessary packet transmission along the network applying the shortest path algorithm after a certain hop-count. Comparatively, TLQoS shows a bit poor performance than TMQoS at low traffic load. The reason is, although it avoids hotspot in routing all packet types, however, the packets might move around more nodes (nodes in the same depth or even in higher depth) in the network that increases the average temperature. However, at high traffic load, TLQoS achieves lower temperature rise than TMQoS due to the less number of retransmissions, and choosing the lower temperature node with more accuracy for Rg data packets employing localized approach. Although, the temperature is chosen as the only routing metric in TARA, still it achieves the highest average temperature rise at high traffic load because of the involvement of more nodes in the network unnecessarily for withdrawal strategy.

As the traffic load increases, the energy consumption also increases in all the protocols as illustrated in Figure 4e. Similar to the temperature rise, ALTR also shows the best performance in energy consumption. The reason is that, although ALTR has lower reliability than QoS-aware protocols but it involves less number of nodes in packet transmission that affects the lower average energy consumption of the nodes. The higher reliability and shorter hop-count path selection also causes to achieve lower energy consumption of QoS aware protocols. Although at low traffic load, TMQoS shows a bit better energy consumption than TLQoS, however, TLQoS outperforms TMQoS at high traffic load due to its superior performance in reliability at that traffic condition. TARA, having poor reliability performance and spreading the packets along more nodes in the network due to withdrawal strategy, exhibits the worst energy consumption performance among all the protocols.

#### 5.3.2. Impact of Bit Error Rate (BER)

**Figure 5:**
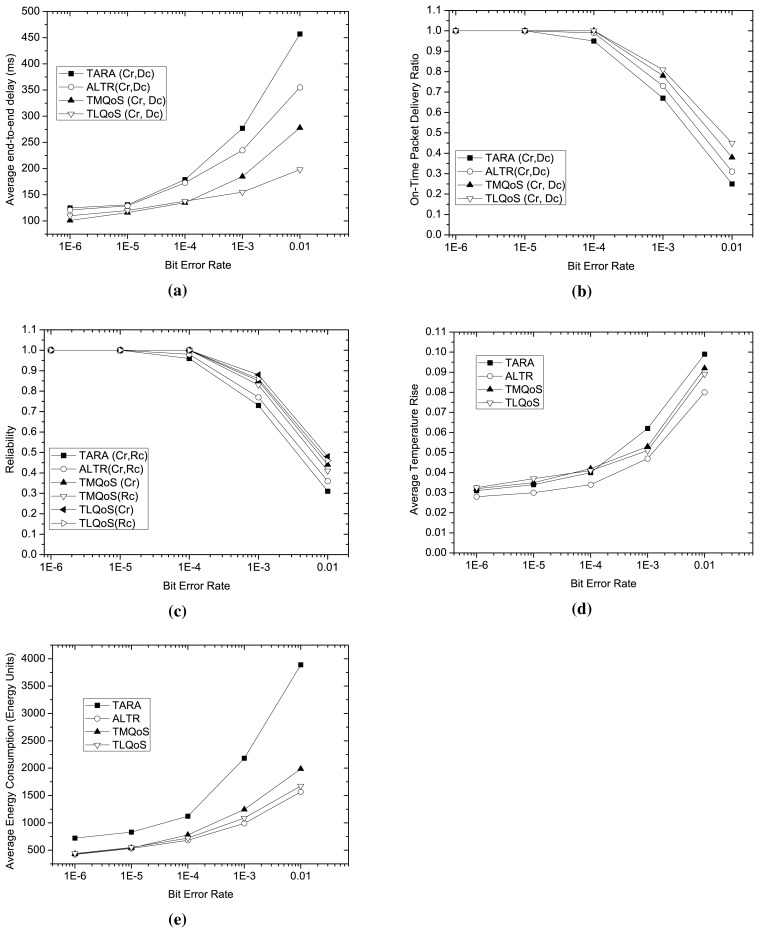
Performance Comparison for different Bit Error Rate. (**a**) Average End-to-End delay varying Bit Error Rate; (**b**) On Time Packet Packet Delivery Ratio varying Bit Error Rate; (**c**) Reliability varying Bit Error Rate; (**d**) Average Temperature Rise varying Bit Error Rate; (**e**) Average Energy Consumption varying Bit Error Rate.

We investigate the impact of wireless link bit error rate on the performance of the protocols considering various metrics as shown in Figure 5. In this study, we set the data generation rate as 1.5 packets per second.

Examining Figures 4 and 5, it is evident that bit error rate influences significantly on the performance metrics compared to traffic load. The reason is that, high bit error rate increases the number of retransmissions that in turn increases the end-to-end latency and diminishes the reliability to a great extent.

As a non-QoS-aware protocol, with the increasing bit error rates, both TARA and ALTR show considerably poor performance in average end-to-end latency, on-time packet delivery ratio and reliability as shown in Figure 5a–c respectively. However, as depicted in Figure 5d,e, ALTR excels all the protocols in average temperature rise and average energy consumption even in high bit error rate due to its selection of least temperature node as a routing metric, and involving less number of nodes in packet transmission through exploiting the shortest path routing after a certain hop-count threshold. Although TARA exploits temperature as its routing metric, but during poor channel condition, it shows the worst performance in temperature rise and average energy consumption as it engages more nodes in packet transmission due to its withdrawal strategy and more retransmissions occurred at higher bit error rate.

Due to the dependence on global view for route selection, TMQoS exhibits poor performance at higher bit error rates than TLQoS in all the performance metrics as shown in Figure 5a–c,e. With the increasing bit error rate, especially when it exceeds 10^−3^, the convergence time increases considerably for TMQoS resulting inaccurate routing information that boosts the end-to-end latency, average temperature rise and average energy consumption, and decreases the on-time delivery ratio and reliability considerably. Because of employing localized and hybrid potential based greedy approach in route selection, TLQoS comparatively shows better performance than TMQoS in all performance metrics during poor channel condition. However, at a very high bit error rate of (>10^−3^), a sharp decline is also observed in case of on-time delivery ratio and reliability performance for TLQoS since, during this channel condition, the packet loss rate for all the nodes increases significantly.

#### 5.3.3. Impact of Delay Deadline

**Figure 6:**
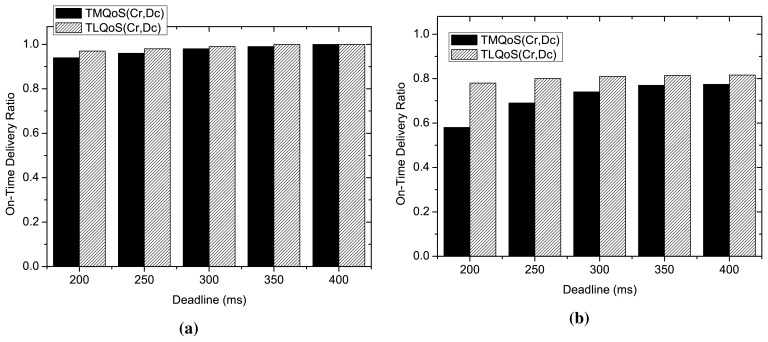
Performance Comparison varying delay deadline. (**a**) On-Time packet delivery ratio varying delay deadline at moderate traffic load; (**b**) On-Time packet delivery ratio varying delay deadline at high traffic load.

This study investigates the impact of delay deadline on on-time packet delivery ratio of the delay sensitive packets for QoS-aware protocols as illustrated in Figure 6. Here, the bit error rate changes randomly ranging from 10^−6^ to 10^−2^. The performance of the protocols is evaluated considering two traffic loads 1.5 pps (Moderate load) and 4 pps (high traffic load).

As the figure shows, at moderate traffic load at 1.5 pps, the delay deadline has little impact on on-time packet delivery ratio for both the protocols. Although during low traffic load, at a strict deadline of 200 ms, a slight difference on on-time delivery ratio of delay sensitive packets is observed between TMQoS and TLQoS, however, both the protocols show almost similar behavior at relaxed deadlines. In contrast, during high traffic load at 4 pps, TLQoS shows much better performance at strict deadline. However, the performance gap for this metric between two protocols reduces gradually with increasing deadline. The situation is characterized by the fact that, during high traffic load, TLQoS shows comparatively better performance than TMQoS due to its localized behavior. Hence, the delay sensitive packets successfully meet the deadline even at a lower value of the deadline.

## 6. Conclusions

In this paper, we have designed TLQoS-a thermal-aware QoS routing protocol to facilitate an effective communication among *in vivo* nodes in WBAN. TLQoS is a distributed protocol that exploits modular design architecture, following several traffic classes. The routing decision is localized exploiting potential-based routing that distinguishes TLQoS from other thermal-aware routing approaches. To evaluate the performance of TLQoS, we perform extensive simulations comparing TLQoS with TARA, ALTR and TMQoS. The results signify that, TLQoS outperforms all the protocols in diverse traffic load situation and channel condition in achieving desired QoS for the relevant traffic types, also maintains moderate temperature rise and energy consumption.

## Figures and Tables

**Table 1:** Simulation Parameters.

**Parameter**	**Value**	**Parameter**	**Value**
EWMA factor, *β*	0.2	EWMA factor, *α*	0.4
Initial Value of *γ*	0.8	*T^thr^*	37.1 ^°^C
*ϵ* at 2 MHz	826	*σ* at 2 MHz	$0.5476\left[\frac{s}{m}\right]$
*P_c_*	0.002	*C_p_*	$3600\left[\frac{J}{kg\cdot {}^{\circ}C}\right]$
*b*	$2700\left[\frac{J}{m^{3}\cdot s\cdot {}^{\circ}C}\right]$	Δ*_t_*	5s
*T_b_*	37 °C	*I*	0.1 *A*
*ρ*	$1040\frac{kg}{m^{3}}$	*K*	$$0.498\left[\frac{J}{m\cdot s\cdot {}^{\circ}C}\right]$$
*Δ*	1m	*HC^max^*	2
*HI^min^*(*TLQoS*)	1s	*HI^max^* (TLQoS)	10 s
HELLO Interval(other protocols)	1s	$D_{up}^{th}$	0.1 s
$R_{up}^{th}$	0.1	$T_{up}^{th}$	0.01 ^°^C
MAC	802.15.4	Bandwidth	100 kbps
Radio Range	2m	Payload Size	256 bits
Retry Limit	5	Simulation Time	1000 s
